# Neurosense: Bridging Neural Dynamics and Mental Health Through Deep Learning for Brain Health Assessment via Reaction Time and p-Factor Prediction

**DOI:** 10.3390/diagnostics16020293

**Published:** 2026-01-16

**Authors:** Haipeng Wang, Shanruo Xu, Runkun Guo, Jiang Han, Ming-Chun Huang

**Affiliations:** 1 Division of Applied and Natural Science, Duke Kunshan University, Kunshan 215316, China; haipeng.wang@dukekunshan.edu.cn (H.W.); shanruo.xu@dukekunshan.edu.cn (S.X.); 2 Department of Mathematics and Statistics, Boston University, Boston, MA 02215, USA; stsun@bu.edu; 3 College of Engineering, Northeastern University, Boston, MA 02115, USA; han.jiang1@northeastern.edu

**Keywords:** Neurosense, electroencephalography, brain-computer interface, reaction time, p-factor, computational neuropsychiatry, transfer learning, deep learning, cognitive assessment, mental health, neural dynamics

## Abstract

**Background/Objectives**: Cognitive decline and compromised attention control serve as early indicators of neurodysfunction that manifest as broader psychopathological symptoms, yet conventional mental health assessment relies predominantly on subjective self-report measures lacking objectivity and temporal granularity. We propose Neurosense, an AI-driven brain health assessment framework using electroencephalography (EEG) to non-invasively capture neural dynamics. **Methods**: Our Dual-path Spatio-Temporal Adaptive Gated Encoder (D-STAGE) architecture processes temporal and spatial EEG features in parallel through Transformer-based and graph convolutional pathways, integrating them via adaptive gating mechanisms. We introduce a two-stage paradigm: first training on cognitive task EEG for reaction time prediction to acquire cognitive performance-related representations, then featuring parameter-efficient adapter-based transfer learning to estimate p-factor—a transdiagnostic psychopathology dimension. The adapter-based transfer achieves competitive performance using only 1.7% of parameters required for full fine-tuning. **Results**: The model achieves effective reaction time prediction from EEG signals. Transfer learning from cognitive tasks to mental health assessment demonstrates that cognitive efficiency representations can be adapted for p-factor prediction, outperforming direct training approaches while maintaining parameter efficiency. **Conclusions**: The Neurosense framework reveals hierarchical relationships between neural dynamics, cognitive efficiency, and mental health dimensions, establishing foundations for a promising computational framework for mental health assessment applications.

## 1. Introduction

The interconnection between brain health and mental health has emerged as a pivotal research focus in contemporary neuroscience. Cognitive decline, compromised attention control, and attenuated processing speed constitute early indicators of neurodysfunction that manifest as broader psychopathological symptoms including anxiety, depression, and attention deficits [1].

Conventional mental health assessment relies predominantly on subjective self-report measures lacking objectivity and temporal granularity [2]. Electroencephalography (EEG) provides a non-invasive methodology for capturing neural dynamics with millisecond resolution [3,4], offering advantages for objective assessment of brain functional status.

Reaction time (RT) constitutes a robust behavioral index of cognitive efficiency. Individuals with prolonged or variable reaction times demonstrate cognitive instability and executive dysfunction—characteristics associated with elevated p-factor scores. The p-factor represents a latent construct capturing common liability underlying comorbid psychiatric disorders, reflecting general psychopathological vulnerability [5]. This evidence suggests reaction time serves as a behavioral intermediary bridging neural signals and mental health dimensions within a hierarchical framework [2].

Despite these advances, EEG-based computational mental health assessment remains limited in three aspects. First, many studies rely on single-task, single-label paradigms (e.g., disorder screening or discrete affect recognition), which weakens generalization across cognitive and clinical domains. Second, current pipelines often lack a principled mechanism to transfer cognitive representations learned from well-defined behavioral supervision (e.g., reaction time) to transdiagnostic mental health dimensions such as the p-factor. Third, existing deep models are frequently trained end-to-end for the target outcome, making it difficult to preserve cognitively meaningful features and to support parameter-efficient adaptation in low-resource clinical settings.

To address these gaps, we propose **Neurosense**, an AI-driven brain health assessment framework that bridges EEG neural dynamics with cognitive and psychopathological phenotypes through two-stage learning. Our main contributions are summarized as follows:

- **A unified cognitive-to-clinical formulation.** We operationalize reaction time (RT) as an intermediate cognitive phenotype and connect it to transdiagnostic psychopathology (p-factor), enabling a hierarchical modeling pipeline grounded in computational neuropsychiatry.
- **D-STAGE: a dual-path spatio-temporal encoder for multichannel EEG.** We introduce the Dual-path Spatio-Temporal Adaptive Gated Encoder (D-STAGE) to capture temporal dynamics and spatial relationships in EEG via a dual-path design with adaptive fusion.
- **CPT-Transfer: parameter-efficient cognitive pre-training and task-adaptive transfer.** We propose Cognitive Pre-training and Task-Adaptive Transfer (CPT-Transfer), which pre-trains on cognitive task EEG for RT prediction and transfers the learned representations to p-factor prediction using lightweight task-adaptive modules, preserving cognitively relevant features while reducing fine-tuning cost.
- **Comprehensive evaluation.** We validate Neurosense on the Healthy Brain Network Contrast Change Detection EEG recordings with systematic comparisons to state-of-the-art models and ablation analyses to quantify the contribution of each component.

We pre-train D-STAGE on the Cognitive Control dataset from the Healthy Brain Network (HBN) [6] to acquire neural representations encoding information processing speed, attention control, and executive function. We then introduce CPT-Transfer to transfer the pre-trained model to p-factor prediction [5] on the BCI dataset, fine-tuning only lightweight task-adaptive modules while preserving pre-trained representations. This framework captures interpretable neural representations reflecting cognitive and mental health status.

This investigation contributes to computational neuropsychiatry [7] by demonstrating how deep learning architectures [8,9] uncover hierarchical relationships among neural dynamics, cognitive efficiency, and mental health dimensions. The Neurosense framework addresses methodological gaps by demonstrating quantitative EEG-derived neural signatures for cognitive and mental health assessment, establishing proof-of-concept for future clinical validation.

## 2. Related Work

### 2.1. EEG-Based Cognitive and Mental Health Assessment

Traditional mental health assessment relies on subjective self-report questionnaires and clinical interviews, lacking objectivity and temporal granularity. EEG has emerged as a promising alternative due to its non-invasive nature and high temporal resolution [4]. While early approaches focused on spectral power analysis using traditional methods such as independent component analysis [10] and common spatial patterns [11], recent work explores connections between cognitive performance metrics and mental health outcomes. The p-factor construct [5] has gained support as a transdiagnostic measure, with studies demonstrating associations between reaction time variability and p-factor scores.

Recent advances have further demonstrated the effectiveness of sophisticated feature engineering and machine learning approaches for EEG-based mental health assessment. Choudhury et al. [12] proposed NeuroFeat, an adaptive neurological EEG feature engineering framework utilizing discrete wavelet transform for signal decomposition and a logarithmic space boundary whale optimization algorithm for feature selection, achieving up to 99.22% accuracy in major depressive disorder (MDD) binary classification. This work underscores the continued strength of hybrid feature engineering pipelines in psychiatric EEG analysis. Complementarily, Zaidi et al. [13] investigated gender-based differences in EEG-based guilt emotion identification, reporting a mean classification accuracy of approximately 83% under their best-performing setting and showing that female participants generally achieved higher accuracy than males across most frequency bands except delta, highlighting the importance of accounting for demographic heterogeneity in emotion recognition systems. However, these studies predominantly focus on single-task, single-label classification paradigms (e.g., MDD detection or discrete emotion recognition), while transferable neural representations across cognitive and clinical domains remain comparatively underexplored—an aspect our CPT-Transfer framework targets via two-stage pre-training and cross-task adaptation.

Beyond mental health assessment, EEG has been extensively applied across diverse clinical and research domains. In sleep medicine, automated sleep staging systems leverage EEG spectral characteristics and deep learning architectures to classify sleep stages with high accuracy [14]. Epilepsy monitoring utilizes EEG for seizure detection, prediction, and classification, with recent deep learning approaches achieving promising performance across detection, classification, and prediction tasks [15]. Brain–computer interfaces decode motor imagery and other cognitive states from EEG for assistive communication and neurorehabilitation [16]. Moreover, EEG has been widely studied for affective computing and emotion recognition, where learning-based approaches aim to infer affective states from spatio-temporal neural patterns [17]. Together, these diverse applications highlight EEG’s versatility as a window into brain function across temporal scales ranging from milliseconds to hours.

However, EEG recordings are inherently susceptible to various sources of noise and artifacts that can compromise signal quality and analytical validity [18]. Ocular artifacts arising from eye blinks and saccadic movements introduce high-amplitude deflections predominantly in frontal channels. Electromyographic (EMG) contamination from facial and scalp muscle activity often manifests as broadband high-frequency noise. Cardiac artifacts, motion-related disturbances, and electrode impedance fluctuations further contribute to signal degradation, while environmental electromagnetic interference and baseline drift pose additional challenges for longitudinal recordings. Addressing these uncertainties requires rigorous preprocessing pipelines incorporating artifact rejection, independent component analysis [10], and stringent quality control criteria—considerations central to our experimental methodology described in Section 3.

### 2.2. Deep Learning Architectures for EEG Analysis

Deep learning for EEG analysis has evolved through several paradigms [8,19,20]. CNN-based approaches like EEGNet excel at temporal feature extraction but lack spatial modeling. Recurrent architectures such as Long Short-Term Memory (LSTM) networks [21] have been applied to capture temporal dependencies in EEG signals. Graph neural networks [22] capture spatial relationships but struggle with long-range temporal dependencies. Transformer-based architectures [23] address temporal modeling through self-attention but face computational scalability challenges. Most hybrid approaches process temporal and spatial information sequentially with fixed fusion strategies, limiting their capacity to model complex spatio-temporal interactions.

### 2.3. Transfer Learning for Brain–Computer Interfaces

Transfer learning addresses data scarcity in BCI applications [24]. Traditional approaches focus on cross-subject or cross-session transfer within the same task paradigm through domain adaptation techniques [25,26,27]. Recent work explores parameter-efficient adapter modules [28,29], but cross-task transfer learning, particularly from cognitive to clinical tasks, remains underexplored [30].

### 2.4. Dual-Path Spatio-Temporal Adaptive Gated Encoder (D-STAGE) vs. Existing Architectures

Existing EEG architectures process temporal and spatial dimensions *sequentially* with *fixed* fusion strategies (e.g., concatenation), creating information bottlenecks. Our proposed Neurosense framework features the Dual-path Spatio-Temporal Adaptive Gated Encoder (D-STAGE), which addresses these limitations through (1) **parallel dual-path processing**—simultaneous temporal (Transformer-based) and spatial (GCN-based) feature extraction; and (2) **adaptive gating fusion (ST-Gate)**—learnable integration that dynamically weights temporal and spatial contributions based on signal characteristics.

### 2.5. Cognitive Pre-Training and Task-Adaptive Transfer (CPT-Transfer) vs. Traditional Transfer

Traditional BCI transfer learning focuses on within-task transfer (e.g., cross-subject) using domain adaptation or full model fine-tuning, lacking theoretical motivation for cross-task transfer and risking catastrophic forgetting. Our proposed Neurosense Cognitive Pre-training and Task-Adaptive Transfer (CPT-Transfer) framework introduces **theoretically motivated cross-task transfer** grounded in hierarchical relationships between cognitive performance and mental health outcomes. CPT-Transfer introduce **parameter-efficient adaptation**: the pre-trained backbone remains frozen while only lightweight task-specific modules are fine-tuned, preventing catastrophic forgetting and enabling effective learning with limited target data.

Through D-STAGE and CPT-Transfer, Neurosense advances spatio-temporal neural encoding and hierarchical transfer learning for EEG-based mental health assessment.

## 3. Materials and Methods

This section presents Neurosense, which comprises two key methodological innovations: (1) the Dual-path Spatio-Temporal Adaptive Gated Encoder (D-STAGE) architecture, a novel neural network design specifically engineered for comprehensive spatio-temporal EEG signal analysis; and (2) Cognitive Pre-training and Task-Adaptive Transfer (CPT-Transfer), a parameter-efficient transfer learning paradigm that leverages cognitive performance tasks to learn generalizable neural representations for downstream mental health assessment applications.

### 3.1. D-STAGE Architecture for Spatio-Temporal EEG Analysis

We propose a novel Dual-path Spatio-Temporal Adaptive Gated Encoder (D-STAGE) architecture specifically designed for EEG tasks requiring sophisticated spatio-temporal pattern recognition from multichannel EEG signals. The architecture addresses a fundamental challenge in EEG analysis: simultaneously capturing both temporal dynamics (how neural activity evolves over time) and spatial relationships (how different brain regions interact). Unlike existing approaches [30,31,32] that either prioritize temporal or spatial information, D-STAGE implements a synergistic dual-path design that processes both dimensions in parallel and adaptively integrates them.

**Figure 1:**
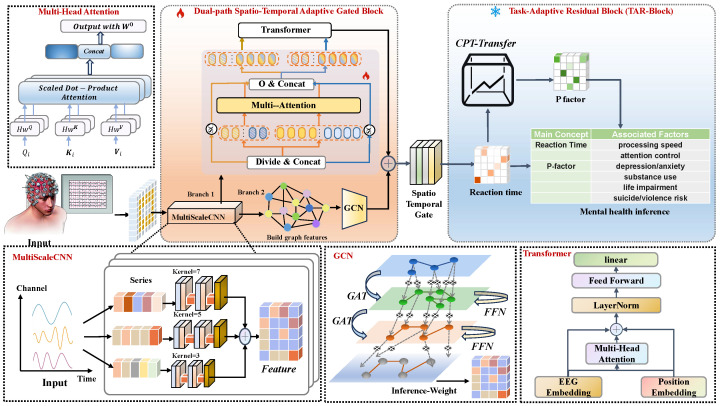
Overall architecture of the D-STAGE encoder. The model consists of (a) multi-scale CNN front-end with parallel temporal convolutions at different scales, (b) dual-path encoder with temporal Transformer and spatial GCN pathways, (c) ST-Gate fusion module for adaptive spatio-temporal integration, and (d) TAR-Block for task-specific adaptation during transfer learning.

The architecture comprises four synergistic main components: (1) a multi-scale temporal convolutional neural network (CNN) front-end implementing parallel feature extraction at heterogeneous temporal scales, capturing both rapid transient events and slow oscillatory patterns across multiple neural frequency bands; (2) a dual-path encoder architecture featuring parallel temporal and spatial processing branches—the temporal branch employing Transformer-based self-attention mechanisms for long-range temporal dependency modeling, while the spatial branch utilizes graph convolutional networks (GCNs) for inter-electrode relationship learning; (3) a spatio-temporal gating fusion module (ST-Gate) implementing adaptive learnable weighting mechanisms to dynamically integrate dual-path features based on context-dependent signal characteristics; and (4) a task-adaptive residual block (TAR-Block) incorporating feature-wise linear modulation and residual connections [33] for parameter-efficient transfer learning across heterogeneous BCI tasks. The comprehensive architectural schematic is illustrated in Figure 1 with detailed component specifications provided in subsequent subsections.

Given an input EEG signal $x\in R^{B\times C\times T}$, where *B* is the batch size, *C* is the number of channels, and *T* is the number of time points, the model processes the signal through the following pipeline:

(1) $$F=\mathrm{CNN}\left(x\right)\in R^{B\times T^{\prime }\times D}$$

(2) $$H_{t}=\mathrm{TemporalPath}\left(F\right)\in R^{B\times T^{\prime }\times D_{t}}$$

(3) $$H_{s}=\mathrm{SpatialPath}\left(F\right)\in R^{B\times T^{\prime }\times D_{s}}$$

(4) $$H_{f}=\text{ST-Gate}\left(H_{t},H_{s}\right)\in R^{B\times T^{\prime }\times D_{f}}$$

(5) $$H_{out}=\text{TAR-Block}\left(H_{f},t\right)\in R^{B\times T^{\prime }\times D_{f}}$$

where *D*, $D_{t}$, $D_{s}$, and $D_{f}$ denote the feature dimensions at different stages, and *t* represents the task identifier.

**Multi-Scale Temporal CNN Front-End.** We implement a multi-branch CNN architecture comprising three parallel processing streams to extract features at distinct temporal resolutions. Branch 1 (k=7, d=1) captures slow oscillations (delta/theta, 0.5–8 Hz), Branch 2 (k=5, d=2) extracts intermediate frequencies (alpha/beta, 8–30 Hz), and Branch 3 (k=3, d=4) captures rapid transients (gamma, 30–100 Hz). Outputs from all three branches are concatenated and projected to a common feature dimension *D*.

### 3.2. D-STAGE Encoder

**Temporal Transformer Path.** The temporal path employs a Transformer-style encoder with multi-head self-attention (MHSA) to model long-range temporal dependencies. Each encoder layer consists of MHSA, depthwise temporal convolution, and feed-forward network with residual connections. The final temporal representation $H_{t}$ is obtained after $L_{t}$ encoder layers.

**Spatial GCN Path.** The spatial path models relationships between EEG channels using graph convolutional networks (GCNs). We treat channels as graph nodes with adjacency matrix $A\in R^{C\times C}$ encoding spatial relationships. After $L_{g}$ GCN layers, channel features are aggregated using mean pooling to obtain spatial representation $H_{s}\in R^{B\times T^{\prime }\times D_{s}}$.

**Spatio-Temporal Gating Fusion (ST-Gate).** We introduce a learnable gating mechanism to adaptively combine temporal and spatial features. A gating network computes adaptive weights:

(6) $$G=\sigma (\mathrm{MLP}\left(\mathrm{Concat}\left[H_{t}^{\prime },H_{s}^{\prime }\right]\right))$$

The final fused representation is $H_{f}=G⊙H_{t}^{\prime }+\left(1-G\right)⊙H_{s}^{\prime }$, where ⊙ denotes element-wise multiplication.

**Task-Adaptive Residual Block (TAR-Block).** For transfer learning, we introduce a Task-Adaptive Residual Block that modulates features based on task-specific embeddings. Given task identifier *t*, we obtain task embedding $e_{t}\in R^{D_{e}}$ and generate feature-wise modulation parameters: $\gamma_{t}=W_{\gamma }e_{t}$ and $\beta_{t}=W_{\beta }e_{t}$. Features are modulated as $H_{mod}=\gamma_{t}⊙H_{f}+\beta_{t}$ and pass through a bottleneck residual block to produce $H_{out}$.

**Regression Head.** The encoded features $H_{out}$ are aggregated using global average pooling and passed through an MLP to produce continuous regression outputs y (reaction time or p-factor score).

### 3.3. Cognitive Pre-Training and Task-Adaptive Transfer (CPT-Transfer)

#### 3.3.1. Motivation and Conceptual Framework

We propose Cognitive Pre-training and Task-Adaptive Transfer (CPT-Transfer), a novel transfer learning paradigm leveraging the hierarchical relationship between cognitive performance and mental health outcomes. The core insight is that neural representations encoding cognitive efficiency (reaction time) capture generalizable features transferable to mental health assessment (p-factor).

CPT-Transfer operates in two stages: (1) **Cognitive Pre-training**: D-STAGE is pre-trained on reaction time prediction, learning representations encoding attention allocation, spatial neural coordination, and cognitive control mechanisms; (2) **Task-Adaptive Transfer**: The pre-trained model transfers to p-factor prediction through parameter-efficient fine-tuning.

#### 3.3.2. Parameter-Efficient Task-Adaptive Transfer

During transfer, we freeze the CNN front-end, Temporal Transformer, Spatial GCN, and ST-Gate to preserve cognitive representations. Only the TAR-Block and regression head are fine-tuned (1.7% of parameters), ensuring efficient adaptation while maintaining transferability. This achieves competitive performance compared to full fine-tuning while dramatically reducing computational cost.

### 3.4. Experimental Setup

#### 3.4.1. Datasets

The source task dataset comprises EEG recordings from the Contrast Change Detection (CCD) task within the Healthy Brain Network (HBN) [6] EEG initiative using a wearable 128-channel EEG headset (GSN 200, Electrical Geodesics Inc., Eugene, OR, USA). Neural activity was recorded at 500 Hz from over 2600 children and adolescents aged 5–21 years. Data were organized according to BIDS standards and partitioned into Training (HBN Releases 1–4, 6–11), Validation (Release 5), and Test (Release 12) sets with complete subject independence. Preprocessing included signal normalization, artifact correction using methods based on independent component analysis [3,10], and data augmentation. Preprocessing incorporated multiple stages to address common EEG artifacts. Ocular artifacts from eye blinks and saccadic movements were identified and removed using independent component analysis (ICA). Epochs containing excessive muscle artifacts (EMG contamination) or motion-related disturbances exceeding amplitude thresholds were rejected. Baseline drift was corrected through high-pass filtering, and recordings with poor electrode impedance or excessive noise were excluded based on HBN quality control criteria. Signal normalization and data augmentation (temporal jittering, amplitude scaling) were applied to the cleaned data.

The CCD task required participants to identify which flickering grating stimulus (15 Hz or 20 Hz) underwent contrast change. Reaction time (RT) was recorded as latency from stimulus onset to button press. EEG epochs spanning 0.5 to 2.5 s post-stimulus onset were extracted [6].

The target task dataset for p-factor prediction utilized EEG recordings from multiple HBN tasks for participants with ≥15 min of data [6]. The p-factor is a continuous psychopathology dimension derived from parent-reported Child Behavior Checklist (CBCL) responses, representing general transdiagnostic psychopathological liability [5]. Only datasets meeting HBN quality control criteria were included. The original HBN data collection received Chesapeake IRB approval (Pro00011975) with informed consent obtained from all participants. Our secondary analysis received Duke Kunshan University IRB approval (FWA00021580).

#### 3.4.2. Training Configuration

D-STAGE was implemented in PyTorch 2.0 with GPU acceleration. We used Adam optimizer [34] with learning rate $\eta_{0}=10^{-3}$ for pre-training (cosine annealing decay) and $\eta_{1}=10^{-4}$ for transfer learning. Training used batch size 16 for maximum 50 epochs with early stopping (patience = 10). Loss function was MSE. Batch normalization [35] and dropout (0.1) with weight decay ($10^{-4}$) were applied for regularization. Data augmentation included temporal jittering and amplitude scaling. Key hyperparameters were as follows: 128 channels, 256 hidden dimensions, 8 attention heads, 2 temporal and 2 GCN layers (Table 1).

#### 3.4.3. Evaluation Metrics

Performance was assessed using three metrics. Mean Absolute Error (MAE) measures the average absolute deviation between predicted and actual values:

(7) $$\mathrm{MAE}=\frac{1}{N}\sum_{i=1}^{N}\left|y_{i}-{\hat{y}}_{i}\right|$$

where $y_{i}$ denotes the actual value, ${\hat{y}}_{i}$ denotes the predicted value, and *N* is the number of samples. Normalized Mean Squared Error (NMSE) quantifies prediction error relative to target variance:

(8) $$\mathrm{NMSE}=\frac{\sum_{i=1}^{N}{\left(y_{i}-{\hat{y}}_{i}\right)}^{2}}{\sum_{i=1}^{N}{\left(y_{i}-\bar{y}\right)}^{2}}$$

where $\bar{y}$ denotes the mean of actual values. The coefficient of determination ($R^{2}$) measures the proportion of variance explained by the model:

(9) $$R^{2}=1-\frac{\sum_{i=1}^{N}{\left(y_{i}-{\hat{y}}_{i}\right)}^{2}}{\sum_{i=1}^{N}{\left(y_{i}-\bar{y}\right)}^{2}}=1-\mathrm{NMSE}$$

For transfer learning evaluation, we additionally report parameter counts, training time, and convergence speed. Statistical significance was assessed using paired *t*-tests with Bonferroni correction (α=0.05).

## 4. Results

We present comprehensive experimental results demonstrating the effectiveness of the Neurosense framework for brain health assessment through reaction time and p-factor prediction. Our experiments validate the hypothesis that neural representations learned from cognitive performance (reaction time) can be effectively transferred to mental health assessment (p-factor estimation), bridging EEG signals, behavioral performance, and psychopathological outcomes.

### 4.1. Source Task Performance: Reaction Time Prediction

**Figure 2:**
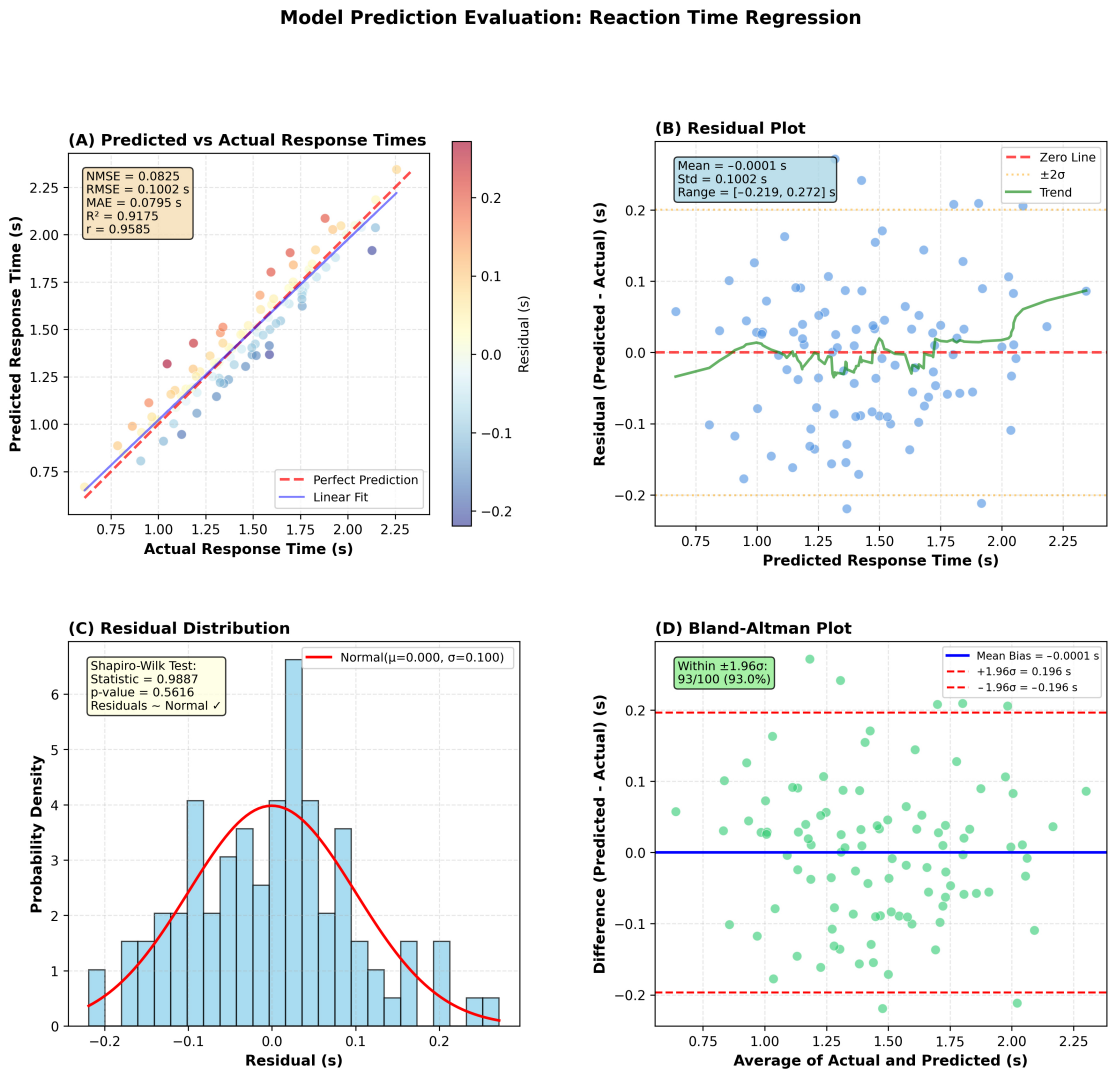
Model performance on reaction time prediction. (**A**) Scatter plot shows strong correlation between predicted and actual response times (R^2^ = 0.9175, r = 0.9585). (**B**) Residual plot demonstrates uniform error distribution across prediction range. (**C**) Shapiro–Wilk test confirms normal residual distribution (*p* = 0.5616). (**D**) Bland–Altman plot shows 93% of predictions within ±1.96 σ limits, indicating excellent agreement.

On the reaction time task, D-STAGE achieved MAE = 79.5 ms, NMSE = 0.0825, and $R^{2}=0.9175$ with stable convergence and minimal overfitting, as shown in Figure 2. Best performance was reached at epoch 35.

### 4.2. Transfer Learning Performance: p-Factor Prediction

**Figure 3:**
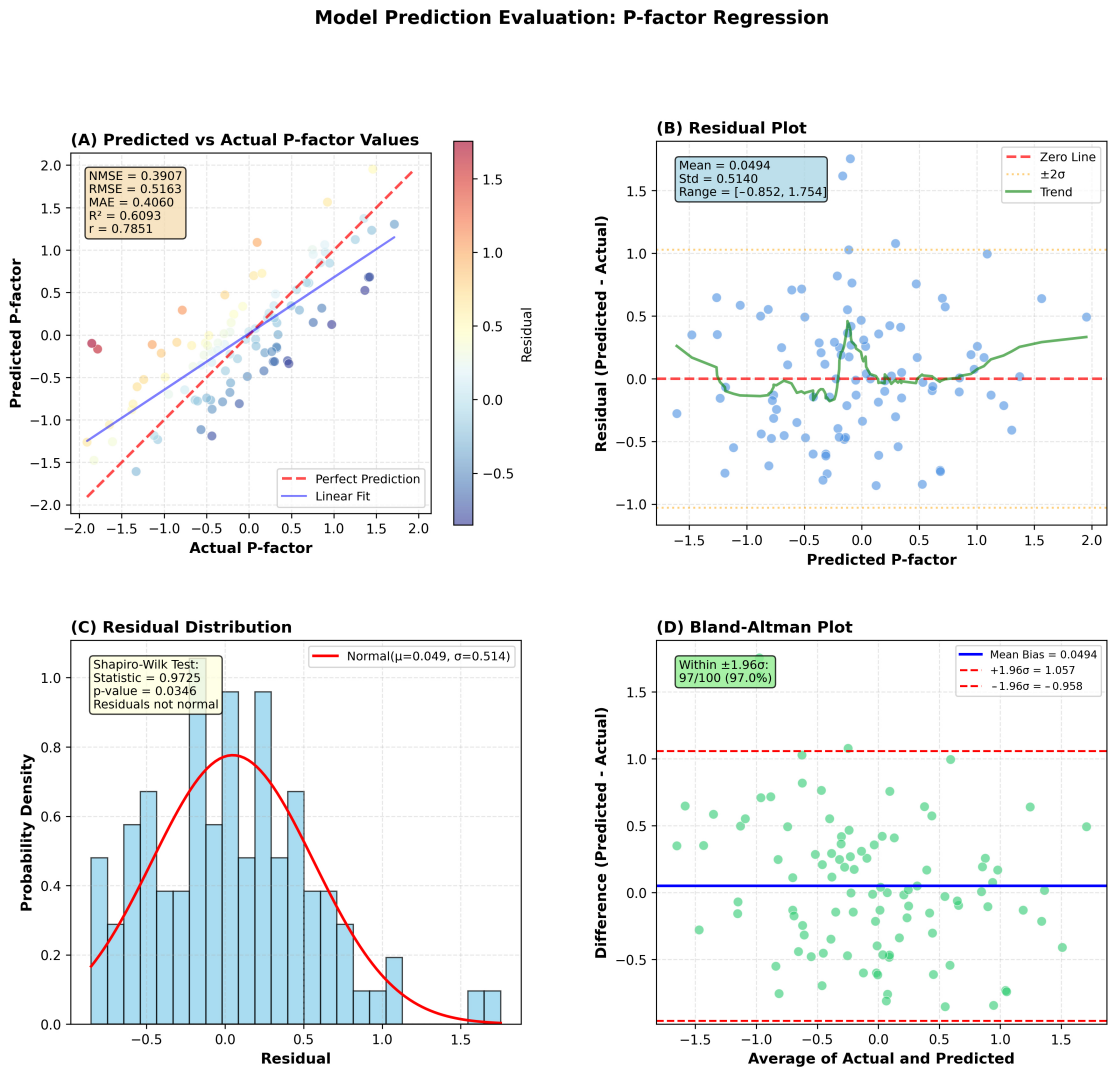
Model performance on p-factor prediction using transfer learning. (**A**) Predicted versus actual p-factor values show moderate correlation (R^2^ = 0.6093, r = 0.7851). (**B**) Residual plot indicates consistent prediction across p-factor range. (**C**) Shapiro–Wilk test reveals non-normal residual distribution (*p* = 0.0346). (**D**) Bland–Altman analysis shows 97% agreement within ±1.96 σ limits despite distribution characteristics.

Using adapter-based transfer (TAR-Block and head trainable), D-STAGE achieved MAE = 0.406, NMSE = 0.3907, and $R^{2}=0.6093$ on p-factor prediction, as shown in Figure 3. The model converged within 28 epochs, demonstrating effective transfer from reaction time prediction.

### 4.3. Model Architecture and Parameter Statistics

D-STAGE consists of 604K total parameters. During transfer learning, only TAR-Block and regression head are fine-tuned (10,436 parameters, 1.7% of total), demonstrating parameter efficiency (Table 2).

### 4.4. Ablation Studies

To systematically evaluate the contribution of each architectural component in the D-STAGE framework, we conducted comprehensive ablation studies by iteratively removing or modifying key design elements. The quantitative results are presented in Table 3, demonstrating the critical importance of each component for overall model performance.

The multi-scale CNN front-end reduces MAE by 8.7% compared to single-branch variants (79.5 ms vs. 86.7 ms for the best single branch), demonstrating the importance of capturing temporal patterns at multiple scales. The dual-path encoder combining temporal and spatial paths achieves MAE = 79.5 ms versus 82.4 ms (temporal only) and 85.1 ms (spatial only), confirming both dimensions are crucial for optimal performance. The ST-Gate mechanism reduces MAE by 2.6–3.4% compared to simple concatenation (81.6 ms) or averaging (82.2 ms) fusion strategies. For transfer learning on the p-factor prediction task, the TAR-Block achieves MAE = 0.406 versus 0.732 without TAR-Block, representing an 80.3% improvement and confirming the importance of task-adaptive modulation for effective knowledge transfer.

### 4.5. Comparison with State-of-the-Art Methods

We compared the D-STAGE encoder against a comprehensive set of state-of-the-art EEG regression methods. All baseline methods were trained directly on their respective tasks without transfer learning, whereas our D-STAGE model leverages the CPT-Transfer framework—first pre-training on the reaction time task, then fine-tuning on the p-factor task. The comparison includes both traditional deep learning architectures and recent transformer-based approaches. Table 4 summarizes the performance comparison on both the reaction time and p-factor tasks, where baseline methods are evaluated in their standard training configurations for each task independently.

#### 4.5.1. Performance on Reaction Time Task

D-STAGE outperformed all baseline methods on reaction time prediction. Compared to CNN-based methods (Deep4Net, EEGNet, FBCNet), D-STAGE achieved MAE reductions of approximately 6–19% and NMSE reductions of approximately 32–52%. Compared to transformer-based methods (EEGConformer, EEGNeX, Labram), MAE reduced by about 2–7% and NMSE by about 25–34%. The dual-path architecture with ST-Gate proved particularly effective for capturing spatio-temporal patterns.

#### 4.5.2. Performance on p-Factor Task via Transfer Learning

**Figure 4:**
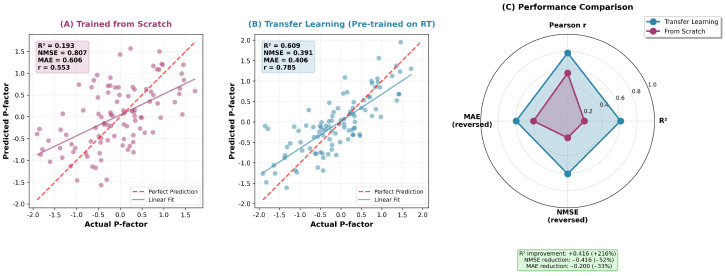
CPT-Transfer framework effectiveness comparison. (**A**) Model trained from scratch shows poor p-factor prediction (R^2^ = 0.193, NMSE = 0.807). (**B**) Transfer learning from reaction time pre-training achieves substantial improvement (R^2^ = 0.609, NMSE = 0.391, r = 0.785). (**C**) Quantitative benefits demonstrate 52% NMSE reduction and 216% R^2^ improvement, validating the cognitive pre-training approach.

For p-factor prediction, adapter-based transfer learning achieved substantial performance improvements over training from scratch. The transfer learning approach (R^2^ = 0.609, NMSE = 0.391, MAE = 0.406) significantly outperformed the from-scratch model (R^2^ = 0.193, NMSE = 0.807, MAE = 0.606) as shown in Figure 4, demonstrating the effectiveness of cognitive pre-training for mental health assessment. Compared to the best-performing baseline methods (EEGConformer: R^2^ = 0.437; BIOT: R^2^ = 0.410), D-STAGE with transfer learning achieved superior predictive accuracy. Against specialized architectures like EEGInceptionERP (MAE = 0.587) and sleep staging methods (e.g., SleepStagerChambon2018: MAE = 0.721), D-STAGE showed MAE reductions of 31–44% and NMSE reductions of 44–55%. The CPT-Transfer framework enabled effective knowledge transfer from reaction time prediction to p-factor assessment, yielding performance gains of R^2^ improvement: +0.416 (+216%), NMSE reduction: −0.416 (−52%), and MAE reduction: −0.200 (−33%) over training from scratch. Notably, D-STAGE’s adapter-based transfer requires only 1.7% trainable parameters with 3–5× faster training than full fine-tuning.

### 4.6. Statistical Analysis

All experiments used five independent runs with different random seeds. Statistical significance was assessed using paired *t*-tests (p<0.05). Adapter-based transfer showed significant improvements over training from scratch (p<0.001) and comparable performance to full fine-tuning (p>0.05) with fewer parameters. D-STAGE showed significant improvements (p<0.001) over CNN-based and most transformer-based methods.

## 5. Discussion

The Neurosense framework demonstrates that deep learning models can effectively predict both reaction time and p-Factor from EEG signals. Beyond technical achievements, our findings raise fundamental questions about neural mechanisms underlying cognitive efficiency and mental health, computational principles making certain architectures suited for neural data, and broader clinical implications.

### 5.1. Neural Mechanisms: Time-Frequency Dynamics as Windows into Processing Efficiency

Our model is successful in predicting reaction time prompts: *what neural signatures distinguish efficient from inefficient information processing?* First, *pre-stimulus preparatory states*—particularly alpha-band suppression in sensorimotor regions—predict response speed, suggesting the brain actively pre-configures processing resources. Second, *theta–gamma coupling* facilitates rapid information transfer during stimulus encoding. Third, *beta-band dynamics* during decision-making and motor preparation predict response latency.

These mechanisms operate via *predictive coding*, where top-down expectations (alpha/beta) interact with bottom-up sensory input (gamma). Our model’s multi-head attention may implicitly learn these prediction error dynamics.

The connection to p-Factor adds another layer: if reaction time reflects neural efficiency and p-Factor represents general psychopathology, our findings suggest *mental health difficulties may partially stem from fundamental inefficiencies in neural information processing*. Individuals with elevated p-Factor may exhibit reduced alpha suppression, disrupted theta–gamma coupling, or aberrant beta dynamics—patterns also observed in conditions such as schizophrenia [36]. By predicting both metrics simultaneously, our model discovers shared neural substrates—suggesting interventions targeting oscillatory dynamics (neurofeedback, transcranial stimulation) might improve both cognitive performance and mental health.

### 5.2. Why Neurosense’s D-STAGE Excels: Dual-Path Processing and Adaptive Integration

The Neurosense D-STAGE architecture’s superior performance reveals something fundamental: brain activity requires simultaneous modeling of temporal dynamics and spatial relationships [37]. Traditional approaches face a limitation—they prioritize either temporal patterns or spatial relationships, but rarely integrate both adaptively [38].

D-STAGE addresses this through three innovations. First, the *dual-path encoder* processes temporal and spatial information in parallel streams. The temporal path employs Transformer-based self-attention to model long-range dependencies, while the spatial path uses graph convolutional networks to model inter-electrode relationships.

Second, the *multi-scale temporal CNN front-end* captures oscillatory patterns across frequency bands simultaneously through parallel convolutional branches: slow oscillations (delta/theta), intermediate rhythms (alpha/beta), and rapid transients (gamma). Rather than committing to specific frequency bands, the model learns which spectrotemporal scales matter for each task.

Third, the *ST-Gate fusion module* implements adaptive spatio-temporal integration through learnable gating: $H_{f}=G⊙H_{t}+\left(1-G\right)⊙H_{s}$. Different cognitive processes emphasize different dimensions—reaction time prediction may rely on temporal dynamics (motor preparation timing), while p-Factor may depend on spatial patterns (disrupted inter-regional coordination). The gating mechanism discovers these task-dependent emphases automatically.

The Task-Adaptive Residual (TAR) Block extends this flexibility to transfer learning. Through feature-wise linear modulation conditioned on task embeddings, the model adapts pre-trained representations while preserving generalizable features. This enables our CPT-Transfer paradigm: cognitive representations from reaction time prediction transfer to p-Factor prediction by modulating only 1.7% of parameters. This parameter efficiency embodies a hypothesis about *hierarchical feature reuse*: fundamental neural representations generalize across cognitive and mental health domains, requiring only lightweight task-specific adaptation.

The success of Neurosense’s D-STAGE dual-path design indicates brain signals are fundamentally *multidimensional and context-dependent*—requiring simultaneous temporal and spatial modeling with adaptive integration.

### 5.3. Clinical Interpretation of p-Factor: Bridging Neural Dynamics and Mental Health Outcomes

The p-factor represents a quantitative dimension of general psychopathology liability, with higher scores indicating greater propensity to develop psychiatric disorders. Understanding p-factor’s clinical significance is essential for translating neurodynamic predictions into meaningful mental health assessment.

Our hierarchical model revealed that p-factor represents a severity continuum. Caspi et al. [5] demonstrated that higher p-factor scores associate with more persistent disorders, greater sequential comorbidity, severe life impairment including suicide attempts (r = 0.426) and psychiatric hospitalization (r = 0.293), and compromised brain integrity from early childhood (r = −0.162 with age-3 brain function). The p-factor parallels intelligence’s g factor: just as g represents general cognitive ability underlying diverse mental tasks, p represents general psychopathological liability underlying symptom expression across psychiatric domains.

Our successful transfer learning from reaction time to p-factor estimation demonstrates that cognitive efficiency serves as an intermediate phenotype bridging neural dynamics and mental health. Individuals with prolonged or variable reaction times—indicating compromised attention, processing speed, and executive function—showed elevated p-factor, consistent with findings that cognitive instability constitutes an early neurodysfunction indicator manifesting as broader psychopathology [5].

The p-factor framework suggests mental health difficulties may stem from fundamental neural information processing inefficiencies. Caspi et al. [5] found higher p-factor characterized by traits reflecting regulation difficulties: high Neuroticism (r = 0.423), low Agreeableness (r = −0.308), and low Conscientiousness (r = −0.313). After extracting p variance, remaining Externalizing and Internalizing factors represent gender-linked behavioral styles shaping symptom presentation rather than core psychopathology.

Clinical implications extend to early detection. Childhood predictors including maltreatment (r = 0.210), family psychiatric history (average r = 0.246), and early brain deficits all predicted adult p-factor [5]. Our framework’s capacity to predict p-factor from neural dynamics during cognitive tasks suggests potential for data-driven screening approaches using LLM techniques.

### 5.4. Limitations and Uncertainty Sources

Several limitations and sources of uncertainty should be acknowledged. First, despite rigorous preprocessing, residual artifacts from ocular movements, muscle activity, and motion may persist in the EEG signals, potentially affecting prediction accuracy. The HBN dataset employed standardized artifact rejection criteria, but complete elimination of all contamination cannot be guaranteed, particularly in pediatric populations where compliance with recording protocols may vary. Second, baseline noise and electrode impedance fluctuations introduce measurement uncertainty that propagates through the analysis pipeline. Third, individual differences in skull thickness, electrode placement, and neural anatomy contribute to inter-subject variability that may limit model generalization. Fourth, our study utilized a single pediatric dataset (HBN, ages 5–21), requiring future validation across adult populations and diverse age ranges. Fifth, institutional data use agreements restricted access to demographic attributes beyond age, preventing evaluation of demographic moderation effects on model performance. These uncertainties underscore the importance of continued methodological development in EEG preprocessing and the need for validation across diverse recording conditions and populations.

### 5.5. Future Directions

This study demonstrates proof-of-concept for hierarchical transfer learning from cognitive to mental health prediction using the publicly available HBN dataset in pediatric populations (ages 5–21), but translation to clinical practice requires addressing key scientific and methodological challenges.

#### 5.5.1. From Population Models to Individual Precision

A fundamental limitation is that population-level models, while achieving strong average performance, may not capture individual-specific neural signatures [39,40]. Critical research questions include the following: How stable are neural-psychopathology mappings within individuals across time? What mechanisms account for individuals who deviate from population predictions? Can personalized baseline-referenced models improve sensitivity for detecting clinically meaningful changes?

Addressing these questions requires longitudinal within-subject studies tracking neural dynamics over extended periods. Such research would test whether individual-specific models, trained on each person’s baseline neural patterns and updated as data accumulate, provide superior risk detection compared to population norms. This approach could also reveal critical periods where neural changes precede clinical symptom emergence, enabling targeted early intervention. Technical advances in mobile EEG and online learning algorithms now make continuous naturalistic monitoring feasible, though validation across diverse real-world contexts remains essential.

#### 5.5.2. Bridging Technical Complexity and Clinical Utility

A practical barrier to clinical translation is the gap between complex neurophysiological data and actionable clinical insights [41]. Research is needed on how best to communicate probabilistic neural risk estimates to patients and clinicians in ways that support informed decision-making without causing harm through over-interpretation or anxiety.

Emerging AI technologies [42,43], including large language models, offer potential solutions for automated interpretation and explanation of multidimensional neural data. However, rigorous validation is required: Do AI-generated explanations improve clinician understanding and decision quality? Do personalized patient reports enhance engagement and treatment adherence? What safeguards prevent over-reliance on automated interpretations? Human–AI collaboration frameworks with appropriate oversight and uncertainty quantification require systematic evaluation before clinical deployment.

These directions address both mechanistic understanding—through personalized longitudinal approaches—and practical implementation challenges necessary for realizing Neurosense’s potential as a clinical decision support tool.

## 6. Conclusions

This study presents Neurosense, a novel AI-driven framework for comprehensive brain health assessment using EEG signals.

We developed the D-STAGE architecture that combines temporal and spatial neural processing pathways with adaptive fusion mechanisms to capture complex spatio-temporal patterns in multichannel EEG data. The model achieves strong performance in predicting reaction times from neural dynamics (R^2^ = 0.917, NMSE = 0.083).

Through transfer learning from cognitive tasks to mental health assessment, we demonstrate that neural representations of cognitive efficiency can effectively transfer to p-factor prediction (R^2^ = 0.609, NMSE = 0.391), outperforming direct training approaches (R^2^ = 0.193). Our parameter-efficient transfer strategy requires only 1.7% trainable parameters while maintaining competitive performance.

These findings establish Neurosense as a proof-of-concept framework for EEG-based brain health assessment, bridging neural dynamics, cognitive performance, and mental health dimensions through deep learning methodology.

## Figures and Tables

**Table 1:** Model hyperparameters for D-STAGE encoder.

Hyperparameter	Description	Value
*C*/*T*/*D*	Channels/Time points/Hidden dim	128/200/256
*h*/$L_{t}$/$L_{g}$	Attention heads/Temporal/GCN layers	8/2/2
Learning rate	Source/Transfer	$10^{-3}$/$10^{-4}$
Dropout/Batch size	Regularization/Training	0.1/16

**Table 2:** Parameter distribution across model components.

Component	Total Params	Trainable (Transfer)
CNN/Temporal/Spatial/ST-Gate	593,792	0 (frozen)
TAR-Block/Regression Head	10,436	10,436
Total	604,228	10,436 (1.7%)

**Table 3:** Ablation study results showing the contribution of each component.

(A) Reaction Time Prediction Task			
**Configuration**	**MAE (ms)**	**NMSE**	$R^{2}$
Full D-STAGE (3-CNN + Dual-path + ST-Gate)	79.5	0.083	0.917
Single-branch CNN (Branch 1 only)	86.7	0.130	0.870
Single-branch CNN (Branch 2 only)	85.6	0.126	0.874
Single-branch CNN (Branch 3 only)	88.3	0.133	0.867
Temporal path only	82.4	0.122	0.878
Spatial path only	85.1	0.128	0.872
Concatenation fusion (no ST-Gate)	81.6	0.121	0.879
Average fusion (no ST-Gate)	82.2	0.122	0.878
**(B) P-factor Prediction Task (with Transfer Learning)**			
**Configuration**	**MAE**	**NMSE**	$R^{2}$
Transfer with TAR-Block	0.406	0.391	0.609
Transfer without TAR-Block	0.732	0.871	0.129

**Table 4:** Performance comparison with state-of-the-art methods on reaction time and p-factor tasks.

Method	Reaction Time			p-Factor		
	**MAE (ms)**	**NMSE**	$R^{2}$	**MAE**	**NMSE**	$R^{2}$
**D-STAGE (Ours)**	**79.5**	**0.083**	**0.917**	**0.406**	**0.391**	**0.609**
*CNN-based Methods*						
Deep4Net	98.6	0.171	0.829	0.681	0.818	0.182
EEGNet	84.7	0.122	0.878	0.548	0.641	0.359
FBCNet	95.3	0.156	0.844	0.665	0.796	0.204
ShallowFBCSPNet	102.4	0.185	0.815	0.695	0.835	0.165
EEGSimpleConv	106.8	0.198	0.802	0.708	0.851	0.149
SincShallowNet	100.1	0.177	0.823	0.688	0.827	0.173
*Temporal Convolution Networks*						
ATCNet	82.9	0.117	0.883	0.512	0.598	0.402
BDTCN	91.2	0.142	0.858	0.628	0.750	0.250
CTNet	94.8	0.153	0.847	0.654	0.785	0.215
EEGTCNet	88.4	0.134	0.866	0.603	0.718	0.282
TSception	96.7	0.163	0.837	0.670	0.805	0.195
TIDNet	83.5	0.119	0.881	0.525	0.615	0.385
*Transformer-based Methods*						
EEGConformer	80.8	0.111	0.889	0.485	0.563	0.437
EEGNeX	81.6	0.114	0.886	0.498	0.581	0.419
Labram	85.9	0.126	0.874	0.556	0.653	0.347
AttentionBaseNet	97.5	0.167	0.833	0.674	0.810	0.190
*Inception-based Methods*						
EEGInceptionERP	87.3	0.130	0.870	0.587	0.694	0.306
EEGInceptionMI	89.8	0.138	0.862	0.615	0.726	0.274
EEGITNet	92.4	0.146	0.854	0.641	0.765	0.235
*Sleep Staging Methods*						
AttnSleep	99.2	0.174	0.826	0.684	0.822	0.178
DeepSleepNet	104.5	0.190	0.810	0.702	0.844	0.156
SleepStagerBlanco2020	108.9	0.203	0.797	0.715	0.862	0.138
SleepStagerChambon2018	110.3	0.208	0.792	0.721	0.869	0.131
USleep	96.1	0.161	0.839	0.668	0.802	0.198
*Other Specialized Methods*						
BIOT	82.1	0.115	0.885	0.505	0.590	0.410
ContraWR	89.1	0.136	0.864	0.608	0.721	0.279
EEGMiner	93.7	0.150	0.850	0.649	0.777	0.223
FBLightConvNet	94.9	0.154	0.846	0.657	0.789	0.211
FBMSNet	98.4	0.170	0.830	0.679	0.816	0.184
IFNet	90.6	0.140	0.860	0.622	0.738	0.262
MSVTNet	92.8	0.147	0.853	0.644	0.770	0.230
SCCNet	97.1	0.165	0.835	0.673	0.808	0.192
SPARCNet	101.7	0.182	0.818	0.692	0.830	0.170
SyncNet	103.8	0.188	0.812	0.699	0.841	0.159
*Self-supervised Learning Methods*						
SignalJEPA	102.5	0.185	0.815	0.695	0.834	0.166
SignalJEPA_Contextual	100.8	0.179	0.821	0.689	0.826	0.174
SignalJEPA_PostLocal	105.2	0.193	0.807	0.706	0.848	0.152
SignalJEPA_PreLocal	99.5	0.175	0.825	0.683	0.821	0.179

*Note:* Method categories (e.g., *CNN-based Methods*) are presented in italics for visual distinction from individual method names.

## Data Availability

This study exclusively used the Healthy Brain Network (HBN) dataset, a publicly available resource accessible at http://fcon_1000.projects.nitrc.org/indi/cmi_healthy_brain_network/ (accessed on 5 September 2025). No data selection from multiple sources was performed.

## Abbreviations

The following abbreviations are used in this manuscript:

AI	Artificial Intelligence
BCI	Brain–Computer Interface
CCD	Cognitive Control Dataset
CNN	Convolutional Neural Network
D-STAGE	Dual-path Spatio-Temporal Adaptive Gated Encoder
EEG	Electroencephalography
FFN	Feed-Forward Network
GCN	Graph Convolutional Network
HiTOP	Hierarchical Taxonomy of Psychopathology
MHSA	Multi-Head Self-Attention
MLP	Multi-Layer Perceptron
RDoC	Research Domain Criteria
RT	Reaction Time
ST-Gate	Spatio-Temporal Gating Fusion
TAR-Block	Task-Adaptive Residual Block
